# Study of quality of life and its determinants in patients after urinary stone fragmentation

**DOI:** 10.1186/1477-7525-8-119

**Published:** 2010-10-19

**Authors:** Mostafa A Arafa, Danny M Rabah

**Affiliations:** 1 King Saud University, King Khalid University Hospital, Princess Al Johara Al Ibrahim for Cancer Research, Prostate Cancer Research Unit, KSA, Riyadh, Saudi Arabia

## Abstract

**Background:**

This study was designed to evaluate the health-related quality of life (HRQOL) of patients who had undergone lithotripsy for treatment of urinary stones and to identify factors that significantly affect the HRQOL of these patients.

**Methods:**

A comparative cross-sectional study was performed at the main university and main Ministry of health hospitals in Riyadh, Saudi Arabia. All patients admitted to the urology service and who underwent lithotripsy for urinary stones during a 9-month period were included in the study. An observation period of 3-15 months following the last treatment was allowed before patients completed the QOL questionnaire. Information on socio-demographic, and medical characteristics, and number and type of lithotripsies were collected. The Medical Outcome Study Short-Form 36-item survey (SF-36) was used to assess HRQoL. For comparison, the HRQoL in an equal number of healthy individuals was investigated; multivariate analysis of variance was used for comparisons between groups.

**Results:**

Compared with healthy subjects, lithotripsy patients had significantly higher mean scores in the different subscales of the SF-36 questionnaire such as physical functioning, vitality, role-physical, role-emotional and mental health, indicating a better HRQOL. Compared with patients who underwent ureteroscopic or extracorporeal shock-wave lithotripsies, those who underwent percutaneous lithotripsy had significantly worse mean scores for all the SF-36 scales, except for body pain. Factors impacting HRQOL of the patients were age, obesity, diabetes mellitus, and stone characteristics such as localization (in the kidney) and recurrence (multiple lithotripsies).

**Conclusions:**

Post-lithotripsy, patients have a favorable HRQOL compared with healthy volunteers. Further prospective studies are warranted to confirm these results owing to the inherent limitations of the cross-sectional design and backward analysis of this study.

## Background

Stone formation in the urinary tract is a common and serious problem encountered in regular urological practice. With a prevalence of more than 10% and an expected recurrence rate of approximately 50%, stone disease has important implications in the healthcare system [1,2]. Extracorporeal shock-wave lithotripsy (ESWL), ureteroscopy (flexible and semirigid) with intracorporeal lithotripsy (URS) and percutaneous nephrolithotripsy (PCNL) are well-established procedures for fragmentation of stones using a lithotriptor. Each modality is associated with advantages and disadvantages, and the choice of modality should be based on well-defined factors, including the type of stone, its location and environment, and other anatomic characteristics [3]. The high prevalence of recurrent stone formation, which in turn is associated with increased morbidity and hospitalization, suggests that stone disease could be a serious health problem that has a significant effect on patients' quality of life (QOL) [1,2,4]. There is an increasing recognition that the selection of therapeutic modalities, irrespective of the type of disease, should be based not only on response rates but also on the effects on the psychological, functional, social and economic life of the patients, including in patients who have undergone lithotripsy for urinary stones [5].

QOL is an estimate of freedom from impairment, disability or handicap [6]. The concept of health-related QOL (HRQOL) is multidimensional and includes psychosocial, physical and emotional status, as well as patient autonomy, and is applicable to a wide variety of medical conditions [7]. To our knowledge, only a few studies have investigated HRQOL in patients undergoing lithotripsy for urinary stones and none has been conducted in Saudi Arabia. Patients with urinary stones represent an ideal group for the investigation of HRQOL owing to specific features of this disease, such as its high prevalence, peak incidence in a socially active generation, severe symptoms and high recurrence rate. Hence, we undertook this study to evaluate the HRQOL in these patients using the SF-36 questionnaire and to investigate the factors that significantly impact HRQOL in these patients.

## Subjects and methods

This was a comparative cross-sectional study conducted over a period of 9 months (January through September 2009) at the main university and main MOH hospitals in Riyadh, Saudi Arabia. All patients (n = 320) admitted to the urology service for surgical intervention for fragmentation of urinary stones during the period of study were invited to participate in the study. The comparator group consisted of an equal number of healthy volunteers selected from the general population or from the individuals or relatives accompanying patients at different outpatient clinics. We included this comparison group owing to the absence of a data base of population 'norms' for our community. Exclusion criteria for subjects in the comparative group included renal disease, urinary stones or any other major disease that could affect the QOL. The two groups were matched in terms of sample size, age and sex. An observation period of 3-15 months following the last lithotripsy was allowed before patients were asked to complete the QOL questionnaire. All patients were interviewed in person by trained personnel at the time of their visits for follow-up examinations. Baseline characteristics included socio-demographic data, medical data and the presence of concomitant health conditions including type 2 diabetes mellitus (DM), hypertension, gout or lower back pain. Data concerning number and type of lithotripsies and size of the stone were retrieved from patient records. The Medical Outcome Study Short-Form 36-item survey (SF-36) [8] was self-administered by both study groups to assess the HRQOL. This tool includes eight scales that assess the following general health measures: physical functioning (PF), role limitations due to physical health problems (role-physical, RP), body pain (BP), general health perceptions (GH), vitality (VT), social functioning (SF), role limitations due to emotional problems (role-emotional, RE), and mental health (MH). Subscale scores are calculated according to standard procedures, yielding score values of 0 to 100, where higher scores indicate better QOL. The study was approved by the institutional review boards of the participating hospitals. All participants provided written informed consent.

### Statistical analysis

Results were expressed as frequencies, means and standard deviations. Data analysis was divided into two parts. Initially, SF-36 subscale scores for the participants were compared across the two main study groups using multivariate analysis of variance (MANOVA). Then, the SF-36 subscales of lithotripsy patients were compared for the three different types of lithotripsies, PCNL, ureteroscopy or EWSL. MANOVA was also used to investigate the impact of different socio-demographic, medical and other related factors on the QOL of the patients. The final multivariate model included lithotripsy type plus all other variables that could affect QOL. The alpha level for the MANOVA test was set at 0.05. Significant statistics (p < 0.05) were followed by post-hoc analyses to determine which subscales were associated with between-group differences, and which specific groups showed significantly differences.

## Results

### Subject demographics

Of the 320 patients invited to participate in the study, 275 patients were enrolled. Forty-five patients discontinued due to non-compliance or loss to follow-up. The age range of patients was 19-90 years, with a mean of 41.45 ± 10.80 years. Nearly two thirds (67%) were male. A majority (92%) were educated to at least secondary school level. Concomitant conditions of hypertension (15%), DM (19%), overweight/obesity (23%), gout (2%) and lower back pain (4%) were noted among the subjects. Included patients had undergone PCNL (97, 35.3%), ureteroscopy (118, 42.9%) or ESWL (60, 21.8%). The observation period after the last lithotripsy before completion of the SF-36 ranged from 3-15 months, with a mean of 9.23 ± 2.4 months. The comparator group (n = 275) consisted of healthy volunteers matched with cases for age and sex. Concomitant conditions of hypertension (8.3%), DM (3.3%), and overweight/obesity (15%) were also noted in this group. There was no significant difference between the two groups in terms of body mass index; 29 ± 4.3 for patients and 28.5 ± 3.2 for controls.

### Health-related quality of life: SF-36 profile

HRQOL was assessed in the two study groups using the SF-36 questionnaire. As seen in Table 1, lithotripsy cases had significantly higher mean scores in the physical functioning, role-physical, vitality, role-emotional and mental health subscales. The greatest differences were observed in mental health (48.96 vs 45.65) and role-emotional subscales (44.78 vs 41.94). There were no significant differences observed in mean scores for general health and social functioning subscales. As regards body pain, lithotripsy cases reported a significantly lower mean score than controls (47.10 vs 49.80).

**Table 1:** Comparison of SF-36 subscales between healthy volunteers (comparator group, n = 275) and patients who had undergone lithotripsy for removal of urinary stones (lithotripsy group, n = 275).

SF-36 Subscale	Group	Mean	SD	F Statistic	P-Value
Physical Functioning (PF)	lithotripsy group	45.43	11.30	10.37	**0.001**
	comparator group	42.15	12.56		

Role-Physical (RP)	lithotripsy group	46.70	7.96	4.46	**0.035**
	comparator group	45.02	10.57		

Body Pain (BP)	lithotripsy group	47.10	8.56	2.67	**0.06**
	comparator group	49.80	9.81		

General Health (GH)	lithotripsy group	49.42	8.39	**0.85**	0.035
	comparator group	49.28	9.33		

Vitality (VT)	lithotripsy group	55.55	10.68	4.89	**0.027**
	comparator group	53.46	9.61		

Social Functioning (SF)	lithotripsy group	44.04	9.73	0.216	**0.642**
	comparator group	44.01	10.86		

Role-Emotional (RE)	lithotripsy group	44.78	10.53	8.127	**0.005**
	comparator group	41.94	12.74		

Mental Health (MH)	lithotripsy group	48.96	10.95	12.65	**0.000**
	comparator group	45.65	10.91		

Table 2 shows a comparative analysis of HRQOL by type of lithotripsy. Patients who underwent PCNL had significantly worse mean scores for all HRQOL domains, except for body pain, while the ESWL patients reported the highest HRQOL scores. The overall test statistic was statistically significant (p < 0.001) for the eight subscales, indicating that there was a correlation between type of lithotripsy and HRQOL.

**Table 2:** Comparison of SF-36 subscales between the patients who had undergone one of the three types of lithotripsy: percutaneous (n = 97), ureteroscopic (n = 118) or extracorporeal shock wave lithotripsy (ESWL, n = 60)

SF-36 Subscale	lithotripsy type	Mean	SD	F Statistic	P-Value
Physical Functioning (PF)	Percutaneous	38.92	13.28		**0.000**
	Ureteroscopy	47.84	8.02	32.82	
	ESWL	51.23	2.72		

Role-Physical (RP)	Percutaneous	43.37	8.13		**0.000**
	Ureteroscopy	47.63	7.78	16.95	
	ESWL	50.24	5.83		

Body Pain (BP)	Percutaneous	48.06	9.74		**0.002**
	Ureteroscopy	45.02	7.42	6.16	
	ESWL	49.24	5.83		

General Health (GH)	Percutaneous	47.50	8.46		**0.007**
	Ureteroscopy	49.85	8.77	5.01	
	ESWL	51.68	6.79		

Vitality (VT)	Percutaneous	51.500	9.45		**0.000**
	Ureteroscopy	55.52	10.33	19.4	
	ESWL	61.76	10.68		

Social Functioning (SF)	Percutaneous	42.45	10.30		**0.000**
	Ureteroscopy	42.93	9.58	9.36	
	ESWL	48.66	7.54		

Role-Emotional (RE)	Percutaneous	39.64	11.31		**0.000**
	Ureteroscoy	45.93	9.44	26.11	
	ESWL	50.82	6.87		

Mental Health (MH)	Percutaneous	44.08	10.09		**0.000**
	Ureteroscopy	49.38	10.46	26.23	
	ESWL	56.01	9.16		

The impact of socio-demographic factors, presence of co-morbidities and other related clinical variables on HRQOL of patients is shown in Table 3. Factors such as age, localization of the stone (in the kidney) and recurrent stones (multiple lithotripsies) significantly affected the HRQOL of patients. Among the concomitant conditions, obesity and DM were found to have a significant impact on HRQOL. It should be noted that the results of the univariate analysis indicated a significant association between poor HRQOL and localization of the stone and recurrent stones in the areas of vitality and mental health. Obesity and DM (type 2) were associated with decreased physical functioning, vitality, role-physical and general health scores. Notably, advanced age significantly reduced HRQOL scores in all domains (data not shown). The Eta square presented in Table 3 reflects the proportion of total variability attributable to each factor.

**Table 3:** Manova general F test to identify factors affecting HRQoL of patients (n = 275) after lithotripsy intervention for treatment of urinary stones.

Factor	F Statistic	P-Value	Partial eta squared
Age	2.27	0.02	**0.09**

Sex	0.91	0.52	**---**

Site of the stone	0.57	0.79	**---**

Location of the stone	3.10	0.003	**0.11**

Presence of stent	1.64	0.31	**----**

Obesity	2.57	0.01	**0.12**

DM (type 2)	3.12	0.001	**0.13**

Recurrent stone (multiple procedures)	2.26	0.02	**0.23**

## Discussion

The prevalence of urinary calculi is estimated to be 1-5% worldwide and it is the third most common problem in urology clinics after urinary tract infection and prostate diseases [9-12]. Moreover, stone disease is one of the most costly diseases worldwide and needs good management and prevention. Many techniques have been proposed for urinary stone management, and several techniques have been developed. Consequently, quantifying clinical results is of critical importance in this non-life threatening disease [13].

Urinary stones can cause a variety of painful symptoms that typically worsen over time, with a high recurrence rate involving about 70% of patients within 20 years of the first renal colic episode and 50% from 4-5 years after the first episode [14]. If symptoms are left unchecked and neglected, these patients are more likely to develop related diseases that will make their health condition more complicated and, in turn, affect their QOL.

Many stones remain asymptomatic for long periods of time, whereas others are associated with symptoms that may necessitate physician evaluation, emergency department visits, hospitalization, or surgical intervention. Although minimally invasive treatments have reduced the morbidity associated with surgical stone management, lifelong medication and/or dietary modification to prevent recurrence is often necessary. In addition, there is an emotional burden associated with living with stones caused by the uncertainty of when or if a stone will become symptomatic [15].

Although many studies have assessed QOL in other urologic disease, few studies have assessed QOL in lithotripsy patients, particularly after treatment. The current study revealed favorable HRQOL scores in seven of the eight SF-36 subscales for post-lithotripsy patients a few months after their last treatment, with significantly higher scores for PF, RP, VT, RE and MH domains compared with the healthy control group (Table 1). Such results may indicate the positive effect of lithotripsy on QOL of patients with this non-life-threatening disease. These patients seem to have a better appreciation of their health, both physically and emotionally, after recovery from urinary stones than before, when their ability to perform work or activities had been impaired due to physical or emotional problems. The improvement in HRQOL may also be explained by the so-called response shift [16]. According to this theoretical model, the often-seen improvement in HRQOL can be a result of an accommodation process that involves changing internal standards and values. It is conceivable that the improved QOL seen in our study is due to such a response shift. On the other hand, lithotripsy patients reported lower BP subscale scores, which may reflect their past experience with pain due to stone formation. Kurahashi et al. [17] reported no significant differences in scores for any scale between lithotripsy patients and healthy volunteers, after an observation period of 3-78 months after the last treatment, in age- and gender-matched Japanese subjects.

A marked change in the strategies for urinary stone removal has been documented. Several types of lithotripsy procedures can be considered depending on clinical parameters and stone characteristics. One of the most important factors that should be considered by clinicians when selecting the lithotripsy procedure for a given patient is the expected changes in HRQOL after the intervention. Our study shows that patients treated by PCNL had significantly lower scores for all domains except body pain, whereas those treated by ESWL had the highest scores (Table 2). Kurahashi et al. found that patients treated by ESWL alone had a significantly higher score for GH perception, whereas no significant differences were detected in the remaining seven scores [17]. On the other hand, this suggested superiority of ESWL was not seen in the study by Mays et al. [18]. Also, according to Rayanal et al., even a minimally invasive technique for stone management is far from being harmless to renal function and can sometimes cause additional symptoms in patients [13].

Patient age, kidney stones, recurrent stones, obesity and DM were the factors with a significant impact on HRQOL in our study. This was particularly true for age, as all domains were associated with poorer QOL, followed by DM and obesity, where four domains were found to be significantly affected (Table 3). Interestingly, indwelling stents and gender did not seem to affect HRQOL scores. Other studies have indicated that pain associated with indwelling stents interfere with daily activities and result in reduced QOL, yet no difference in QOL and urinary symptoms and pain were detected using stents of different size [19-21]. The study of Penniston and Nakada reported that women scored significantly lower than men for all domains [22]. The results of the current study are in agreement with those of previous studies, namely that quality of life impairments are magnified in patients with associated co-morbidities such as DM, obesity, hypertension, musculoskeletal disorders, and depression [22,23]. However, in the present work, only diabetes and obesity were found to have a significant impact on QOL. Obesity and DM type 2 are quite prevalent in Saudi Arabia, which may explain their influence on patient HRQOL [24,25]. Another factor that had a negative impact on patient HRQOL is recurrent stones, likely due to the effects of recurrent symptoms due to renal colic, hence recurrent surgical procedures that could affect patient QOL [23,26].

### Potential limitations of the study

First, the follow-up period chosen in this study (3-15 months) was relatively short, because 50% of cases are known to recur within 5 years of the initial stone event. Secondly, being a cross-sectional, retrospective study, we could not evaluate the baseline HRQOL before stone development. This may be rectified in a future study through periodic follow-up and regular assessment of patient QOL. Third, stressful life events were not assessed in this study. It is well known that such events can influence HRQOL and negatively impact patient perception of health status. Finally, Saudi population 'norms' are not available, which limited the calculation of summary composite scores.

## Conclusions

Patients' expectations and QOL are paramount in the current era of clinical practice. Although invasive procedures often have a negative effect on HRQOL, lithotripsy patients, after a reasonable period of recovery from surgical procedures, had a favorable HRQOL compared with a healthy control population. This is particularly important for this non-life-threatening disease, owing to the factors and surgical interventions that could have a negative influence of patients' QOL. Further longitudinal and prospective studies are warranted to further assess the impact of different factors and surgical interventions on QOL and to overcome the inherent disadvantages associated with backward studies.

## Competing interests

The authors declare that they have no competing interests.

## Authors' contributions

MA Participated in the design of the study, writing the paper and performed the statistical analysis. DR Participated in its design and coordination. Both authors read and approved the final manuscript.
